# Recurrence of Benign Vocal Fold Lesions: Risk Factors and an Exploratory Comparison of Cold-Instrument and CO_2_ Laser Surgery in a Retrospective Cohort

**DOI:** 10.3390/medicina62091779

**Published:** 2026-09-16

**Authors:** Alexandru Vlase, Octavian Dragos Palade, Dragoș Munteanu, Ionut Andrei Roman, Stefan Moscalu, Ana Doina Roxana, Andreea Rusescu, Ruxandra Oana Aliuș, Catalina Voiosu

**Affiliations:** 1 Faculty of Medicine, “Grigore T. Popa” University of Medicine and Pharmacy, 700115 Iași, Romaniadragos.munteanu@umfiasi.ro (D.M.);; 2 ENT Department, Clinical Hospital “Căi Ferate” Iași, 700506 Iași, Romania; 3 Emergency Clinical Hospital “Sf. Spiridon” Iași, 700111 Iași, Romania; 4 Internal Medicine Department, Clinical Hospital “Căi Ferate” Iași, 700506 Iași, Romania; 5 Faculty of Medicine, “Carol Davila” University of Medicine and Pharmacy, 011172 Bucharest, Romania; andreea.rusescu@umfcd.ro (A.R.);; 6 “Prof. Dr. Dorin Hociotă” Institute of Phonoaudiology and Functional ENT Surgery, 050751 Bucharest, Romania

**Keywords:** benign vocal fold lesions, vocal fold polyp, vocal fold nodule, recurrence, persistent disease, CO_2_ laser, cold instruments, microlaryngoscopy, dysphonia, phonosurgery

## Abstract

*Background and Objectives:* Benign vocal fold lesions (BVFLs) are common causes of persistent dysphonia, but recurrence estimates are influenced by lesion type, outcome definition, and follow-up. We evaluated recurrence after documented lesion resolution and explored clinical factors and surgical technique. *Materials and Methods:* We performed a single-center retrospective cohort study of 214 adults with a first documented BVFL diagnosis between January 2022 and December 2024. Clinical records were reviewed through January 2026. Recurrence was defined as lesion reappearance after documented resolution at the initial post-treatment laryngeal assessment; persistent lesions were classified separately. *Results:* Follow-up was documented in 142 patients (66.4%). Initial lesion resolution was documented in 135 patients, of whom 23 developed recurrence (17.0%); 7 patients had persistent disease at the initial post-treatment assessment. No evaluated demographic or clinical factor was significantly associated with recurrence in univariate analyses. In the secondary exploratory surgical analysis, recurrence occurred in 7/29 patients treated with cold instruments (24.1%) and 3/27 treated with CO_2_ laser (11.1%; OR 2.55, 95% CI 0.58–11.08; Fisher exact *p* = 0.299). *Conclusions:* Documented recurrence after initial lesion resolution occurred in 17.0% of patients. The surgical technique comparison was underpowered and unadjusted and does not establish superiority of either technique. Variable follow-up and differential attrition should be considered when interpreting the recurrence proportion.

## 1. Introduction

Benign vocal fold lesions (BVFLs) comprise a heterogeneous group of non-malignant laryngeal disorders, including vocal fold nodules, polyps, Reinke edema, intracordal cysts, granulomas, vascular lesions, and sulcus-related abnormalities [1,2]. Although benign, these conditions can cause persistent dysphonia, vocal fatigue, occupational impairment, and reduced quality of life, particularly in patients with high vocal demands [3].

The pathogenesis of BVFLs is multifactorial. Mechanical phonotrauma and maladaptive voice use are central mechanisms for many lesions [4]. Reflux-related mucosal irritation may contribute to selected BVFLs [5], while smoking and other irritant exposures have also been associated with benign vocal fold pathology [6]. Polyps and nodules arise predominantly within the superficial lamina propria and may share reactive stromal and vascular changes [3,4]. A standardized nomenclature for benign mid-membranous vocal fold lesions has been proposed to improve consistency in clinical classification [7]. Management is individualized and may include vocal hygiene, treatment of contributory conditions, voice therapy when available, and phonosurgical intervention [1,8,9].

Recurrence after treatment remains clinically relevant, but reported estimates vary substantially across studies because lesion types, recurrence definitions, adjunctive therapy, and observation periods differ [2,10,11]. In retrospective cohorts, the distinction between persistent disease and recurrence after documented resolution is particularly important: a lesion that never resolves should not be classified as recurrent. Unequal follow-up also creates different opportunities for recurrence detection and may bias crude recurrence proportions.

The objectives of this study were to describe the demographic and clinical profile of adults with BVFLs, evaluate factors associated with recurrence after documented initial lesion resolution, describe the laterality of the dominant/index lesion, and perform a secondary exploratory comparison of recurrence after cold-instrument microlaryngeal surgery versus CO_2_ laser surgery.

## 2. Materials and Methods

### 2.1. Study Design and Setting

This was a single-center retrospective observational cohort study conducted in the ENT Department of Clinical Hospital “Căi Ferate” Iași, a tertiary otolaryngology department affiliated with “Grigore T. Popa” University of Medicine and Pharmacy, Iași, Romania. Consecutive eligible adults with a first documented diagnosis of a benign vocal fold lesion between January 2022 and December 2024 were identified. Clinical follow-up data were extracted from the medical records through January 2026.

### 2.2. Participants

The cohort included 214 adults aged 18 years or older with a clinical diagnosis of a benign vocal fold lesion based on otolaryngologic assessment and routine laryngeal examination. Only patients presenting with a first documented BVFL diagnosis were included; patients with a previous benign vocal fold lesion or prior phonosurgery were not included in the cohort. Additional exclusion criteria were malignant or premalignant laryngeal lesions, previous laryngeal carcinoma, and insufficient diagnostic information. Follow-up availability was not an inclusion criterion for the descriptive cohort, because one objective was to characterize all consecutive eligible patients during the study period.

### 2.3. Treatment and Surgical Technique

Treatment was selected according to lesion characteristics, symptom severity, vocal requirements, and clinician judgment. Conservative management included vocal hygiene recommendations and medical treatment of documented contributory conditions. Formal voice therapy services were not systematically available in the local study setting. Some patients reported independently obtaining voice training or voice therapy interventions, but this information was not consistently documented and could not be quantified or analyzed.

Surgical treatment was performed under general anesthesia and suspension microlaryngoscopy using either conventional cold instruments or a CO_2_ laser. Technique selection depended on lesion characteristics, surgeon preference, equipment availability, and intraoperative assessment. Phonomicrosurgical principles, including preservation of the superficial lamina propria and avoidance of injury to the vocal ligament, were applied whenever feasible.

CO_2_ laser procedures were performed using a Lumenis 40C CO_2_ laser system (Lumenis Ltd., Yokneam, Israel; wavelength 10.6 μm). Depending on lesion characteristics, the laser was used in continuous-wave or repetitive-pulse mode, with power settings of 2–4 W and a pulse duration of 0.4 s when repetitive-pulse mode was selected.

### 2.4. Variables and Outcome Definitions

Extracted variables included age, sex, urban or rural residence, clinical diagnosis, laterality of the dominant/index lesion, documented professional voice use, smoking status, documented regular/chronic alcohol use, documented gastric reflux disease or laryngopharyngeal reflux (LPR), main symptom, treatment modality, surgical technique, histopathology when available, follow-up duration, and recurrence status. Reflux status was based on the diagnosis recorded in the medical record and was not independently confirmed by objective reflux testing. Professional voice use, smoking, and alcohol exposure were analyzed as categorical variables because quantitative exposure data were not consistently available.

The laterality variable represented the side of the dominant/index lesion documented in the clinical database. In patients with vocal fold nodules, when one side was considered the dominant lesion and the contralateral finding was described as a reactive/contact lesion, only the dominant side was entered in the laterality field. Consequently, the laterality variable should not be interpreted as a complete measure of all bilateral mucosal abnormalities.

Follow-up records were reviewed to distinguish persistent disease from recurrence. Recurrence was defined as documented reappearance of a benign vocal fold lesion after documented resolution at the initial post-treatment laryngeal examination, which was typically performed 4–8 weeks after treatment. A lesion that remained present at the initial post-treatment assessment without documented resolution was classified as persistent disease rather than recurrence. No fixed prospective disease-free interval had been predefined because of the retrospective design. Recurrence analyses were therefore restricted to patients with documented initial lesion resolution. Patients without documented follow-up were considered to have unknown outcome status and were not included in recurrence analyses.

### 2.5. Follow-Up Assessment and Data Closure

Follow-up was not protocolized and reflected routine clinical practice. Follow-up duration was calculated from the date of initial treatment to the last documented laryngeal examination. No predefined minimum follow-up duration was required beyond the initial post-treatment assessment. Some patients returned for only 3 or 6 months and did not attend subsequent visits, whereas others continued follow-up for up to 24 months. Patients contributed observation time only through their last documented evaluation. Therefore, “no documented recurrence” indicates absence of recurrence during the available observation period and does not imply that recurrence could not have occurred after follow-up ended. The database was closed in January 2026.

### 2.6. Statistical Analysis

Continuous variables were summarized as mean ± standard deviation (SD) and median with range when appropriate. Categorical variables were summarized as counts and percentages. The primary recurrence analysis included the 135 patients with documented initial lesion resolution; the seven patients with persistent disease were analyzed separately and were not included in the recurrence denominator. Age was compared using Welch’s *t*-test, and binary categorical variables were compared using Fisher’s exact test. Odds ratios (ORs) with 95% confidence intervals (CIs) were reported as exploratory effect estimates. The association between dominant/index lesion laterality and recurrence was evaluated using the Fisher–Freeman–Halton exact test because of sparse cell counts.

Treatment modality (conservative versus surgical) and surgical technique (cold instruments versus CO_2_ laser) were compared using Fisher’s exact test. Because lesion type may confound technique selection, a polyp-only sensitivity analysis was performed among the followed surgical patients. Follow-up duration was compared between relevant groups using the Mann–Whitney U test because follow-up intervals were discrete and non-standardized. Baseline characteristics of patients with versus without documented follow-up were compared using Welch’s *t*-test for age and Fisher’s exact tests for binary variables; the overall diagnosis distribution was compared using the Fisher–Freeman–Halton exact test because several cells were sparse.

A conventional multivariable logistic model was not fitted. After distinguishing persistence from recurrence, only 23 recurrence events were available in the overall analytic cohort and 10 in the followed surgical subgroup. Including the full set of candidate predictors in a conventional multivariable model would have created a substantial risk of overfitting and unstable estimates, and no smaller predictor set had been prespecified. Accordingly, the association analyses were retained as exploratory univariate analyses. Statistical analyses were performed using IBM SPSS Statistics for Windows, version 26.0 (IBM Corp., Armonk, NY, USA). All tests were two-sided, and *p* < 0.05 was considered statistically significant.

## 3. Results

### 3.1. Cohort Characteristics and Patient Flow

The total cohort comprised 214 patients. Mean age was 49.6 ± 16.9 years and median age was 52 years (range, 18–80 years). Men accounted for 138 patients (64.5%), and 173 patients (80.8%) lived in urban areas. Professional voice use was documented in 85 patients (39.7%), current smoking in 77 (36.0%), chronic alcohol use in 39 (18.2%), and reflux disease/LPR in 112 (52.3%). Surgical treatment was performed in 103 patients (48.1%). Baseline cohort characteristics are summarized in Table 1.

**Figure 1:**
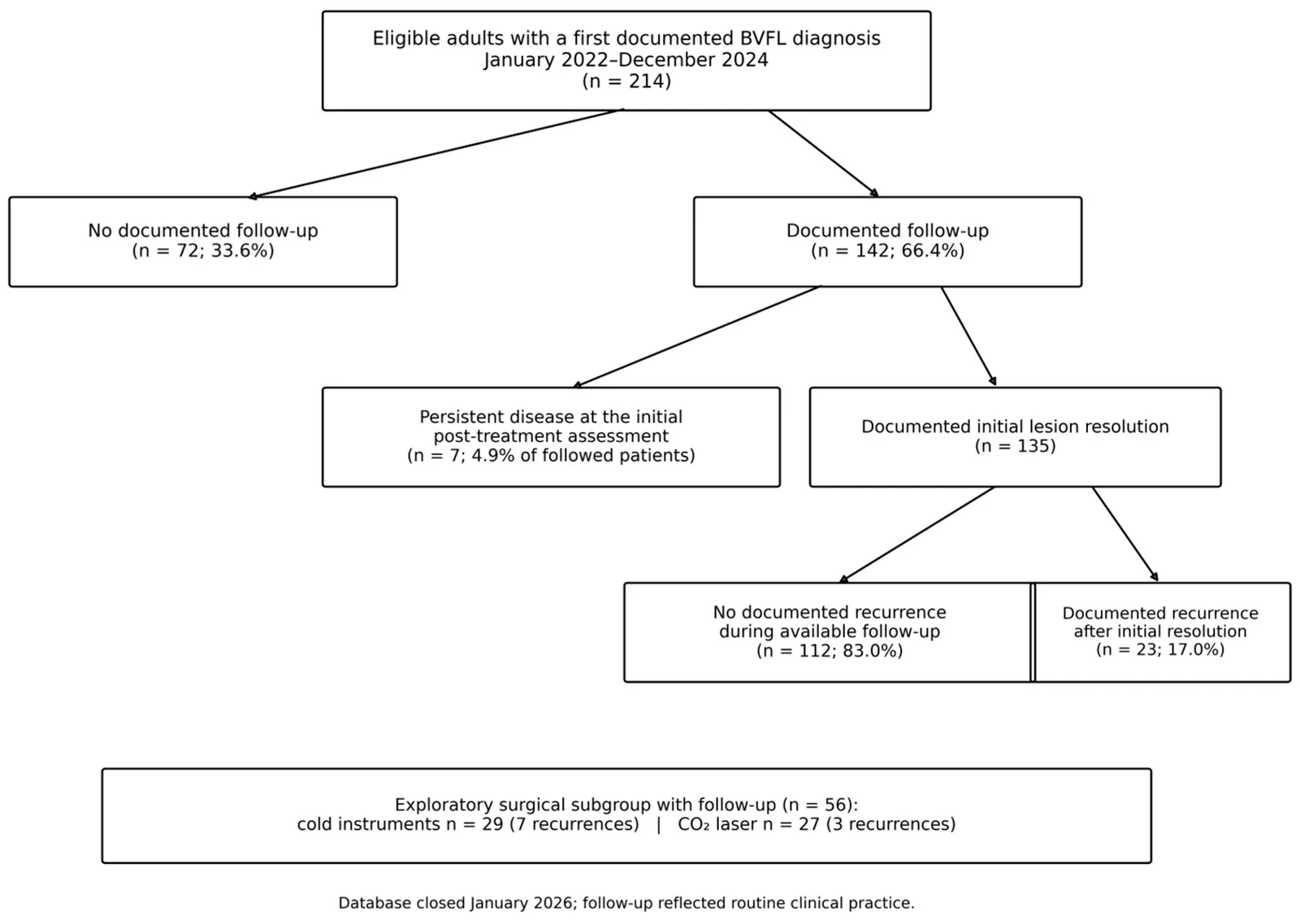
Patient flow and recurrence classification. Persistent disease indicates a lesion remaining at the initial post-treatment assessment; documented recurrence after initial resolution required documented initial lesion resolution followed by later reappearance. The surgical technique comparison was a secondary exploratory analysis.

Documented follow-up was available for 142 patients (66.4%), whereas 72 (33.6%) had no documented follow-up. Among the 142 followed patients, 135 (95.1%) had documented initial lesion resolution and entered the recurrence analytic cohort. Seven patients (4.9% of followed patients) had persistent disease at the initial post-treatment assessment and were analyzed separately. Histopathological examination was available for all 103 surgically treated patients and confirmed benign pathology. Patient flow and outcome classification are shown in Figure 1.

### 3.2. Diagnostic Distribution and Dominant/Index Lesion Laterality

**Figure 2:**
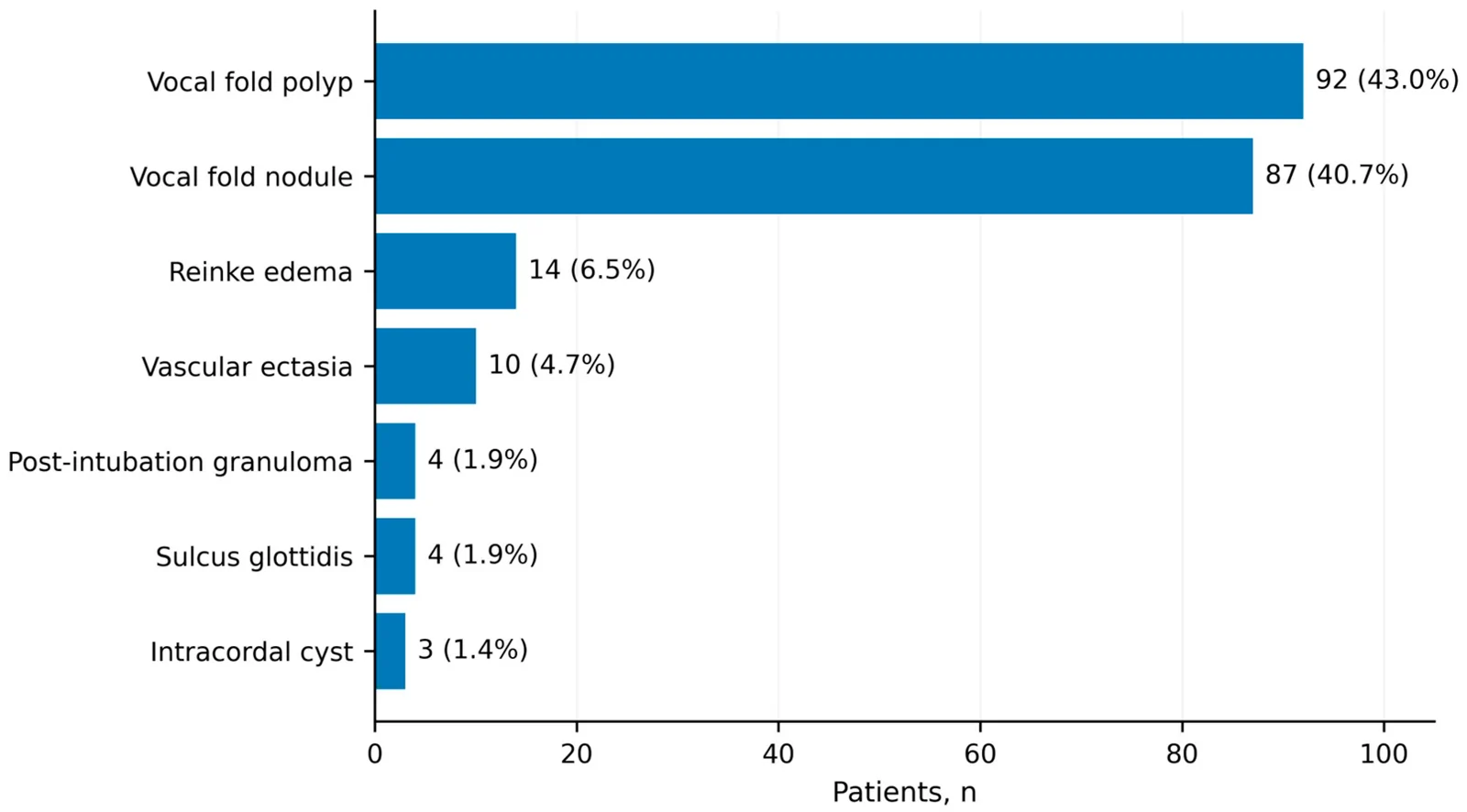
Distribution of benign vocal fold lesion diagnoses in the total cohort (n = 214). Values are presented as n (%).

Vocal fold polyps and nodules accounted for most diagnoses. Polyps were recorded in 92 patients (43.0%) and nodules in 87 (40.7%). Reinke edema, vascular ectasia, post-intubation granuloma, sulcus glottidis, and intracordal cysts were less frequent. The diagnostic distribution is summarized in Table 2 and Figure 2.

The dominant/index lesion was coded on the left in 102 patients (47.7%), on the right in 94 (43.9%), and as bilateral in 18 (8.4%). These figures represent database coding of the dominant/index lesion rather than the prevalence of all contralateral reactive findings. In particular, contralateral reactive/contact changes accompanying a dominant vocal fold nodule were not coded as bilateral primary disease. Laterality data are summarized in Table 3.

### 3.3. Recurrence, Persistence, and Clinical Factors

Among the 135 patients with documented initial lesion resolution, recurrence was documented in 23 (17.0%), while 112 (83.0%) had no documented recurrence during their available follow-up. Persistent disease was observed in seven additional patients and was not counted as recurrence. Mean follow-up among all 142 followed patients was 9.1 ± 4.8 months, with a median of 9 months (range, 3–24 months).

Recurrence differed descriptively across lesion types but subgroup numbers were small. Recurrence occurred in 10/53 polyps (18.9%), 11/61 nodules (18.0%), and 2/8 cases of Reinke edema (25.0%). No recurrence after documented initial resolution was observed among patients with vascular ectasia, post-intubation granuloma, sulcus glottidis, or intracordal cyst. Five of the seven persistent lesions were nodules, one was a post-intubation granuloma, and one was sulcus glottidis. Lesion-specific recurrence is summarized in Table 4.

No evaluated demographic or clinical variable reached statistical significance in univariate analysis (Table 5). Current smokers had a numerically higher recurrence proportion than non-smokers (11/51, 21.6% versus 12/84, 14.3%), but the estimate was imprecise (OR 1.65, 95% CI 0.67–4.08; *p* = 0.346) and does not provide evidence of a smoking effect in this cohort. Recurrence occurred in 10/56 surgically treated patients (17.9%) and 13/79 conservatively treated patients (16.5%; OR 1.10, 95% CI 0.45–2.73; Fisher exact *p* = 0.821).

### 3.4. Exploratory Surgical Technique Analysis

Of the 103 surgically treated patients in the total cohort, 55 (53.4%) underwent cold-instrument microlaryngeal surgery and 48 (46.6%) underwent CO_2_ laser surgery. Among the 56 surgically treated patients with documented follow-up, 29 had been treated with cold instruments and 27 with CO_2_ laser. All 56 had documented initial lesion resolution and were therefore included in the recurrence analysis.

The characteristics of the actual surgical analytic cohort are shown in Table 6. The groups were similar in age and sex distribution, but lesion composition differed substantially: all five followed vascular ectasia cases and all four followed Reinke edema surgical cases were in the cold-instrument group, whereas 26 of 27 patients in the CO_2_ group had vocal fold polyps. This imbalance illustrates substantial potential for confounding by indication.

**Figure 3:**
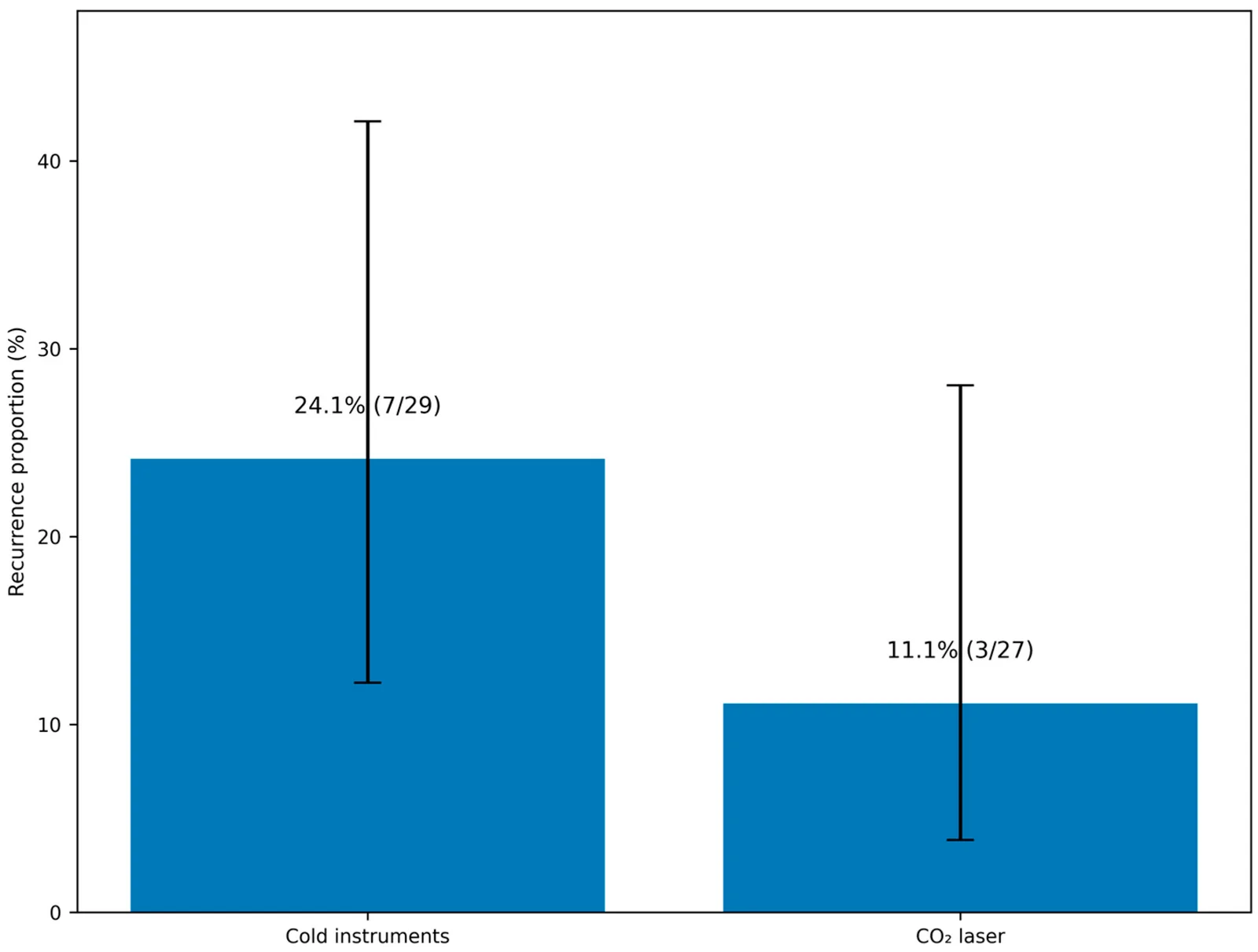
Documented recurrence according to surgical technique among the 56 followed surgical patients. Bars show recurrence proportions and error bars show 95% Wilson confidence intervals. Fisher exact *p* = 0.299.

Recurrence was documented in 7/29 patients treated with cold instruments (24.1%) and 3/27 treated with CO_2_ laser (11.1%). The difference was not statistically significant (OR for cold instruments versus CO_2_ laser 2.55, 95% CI 0.58–11.08; Fisher exact *p* = 0.299). In a polyp-only sensitivity analysis, recurrence occurred in 5/19 cold-instrument cases (26.3%) and 3/26 CO_2_ laser cases (11.5%; OR 2.74, 95% CI 0.57–13.27; *p* = 0.253). The direction of the point estimate was similar, but the confidence interval remained wide and the analysis was underpowered. These results are summarized in Table 7 and Figure 3.

### 3.5. Follow-Up Duration, Laterality, and Attrition

Observation time was not uniform. Patients with documented recurrence had longer available follow-up than patients with no documented recurrence (median 12 versus 6 months; Mann–Whitney U *p* = 0.016), consistent with a greater opportunity for recurrence detection during longer observation. Among patients with documented initial lesion resolution, follow-up duration did not differ significantly between surgical and conservative management (*p* = 0.114) or between the cold-instrument and CO_2_ laser surgical groups (*p* = 0.247). Follow-up-duration comparisons are summarized in Table 8.

Among the 135 patients in the recurrence analytic cohort, recurrence occurred in 13/58 patients with a left-sided dominant/index lesion (22.4%), 8/66 with a right-sided dominant/index lesion (12.1%), and 2/11 with bilateral index disease (18.2%). The association was not statistically significant (Fisher–Freeman–Halton exact *p* = 0.305). Because the laterality variable represented the dominant/index lesion, this analysis should not be interpreted as a comparison of all unilateral versus bilateral mucosal abnormalities.

Comparison of baseline characteristics between patients with and without documented follow-up showed evidence of differential attrition (Table 9). Patients without documented follow-up were more often male (76.4% versus 58.5%; *p* = 0.010) and more often underwent surgical treatment (65.3% versus 39.4%; *p* < 0.001). Age, smoking, professional voice use, alcohol use, reflux status, urban residence, and surgical technique distribution among surgical patients did not differ significantly. The overall diagnosis distribution differed between groups (Fisher–Freeman–Halton exact *p* = 0.042); lesion-specific inferential comparisons were not performed because of sparse cells.

## 4. Discussion

This retrospective cohort study evaluated recurrence after benign vocal fold lesions in 214 adults and incorporated surgical technique as a secondary exploratory treatment-related variable. Persistent disease was separated from recurrence according to documented initial lesion resolution. Among 135 patients with documented initial lesion resolution, 23 developed recurrence during the available observation period, corresponding to 17.0%. Seven additional patients had persistent disease at the initial post-treatment assessment. No evaluated demographic or clinical factor was significantly associated with recurrence in univariate analyses.

The distinction between persistence and recurrence materially improved outcome definition. Five of the seven persistent lesions were vocal fold nodules, a lesion type for which incomplete response to conservative management can easily be mistaken for recurrence if documented resolution is not required. The recurrence analysis therefore used documented initial resolution as an eligibility requirement for the recurrence denominator. Nevertheless, no standardized minimum disease-free interval had been predefined, and the recurrence endpoint remains dependent on the quality and timing of retrospective clinical documentation.

Polyps and nodules accounted for more than four-fifths of the cohort. Among patients with documented initial resolution, recurrence proportions were similar for polyps (18.9%) and nodules (18.0%) and numerically higher for Reinke edema (25.0%), although the Reinke subgroup was very small. These descriptive findings should not be interpreted as comparative lesion-specific risks. Prior studies have similarly reported substantial heterogeneity in recurrence according to lesion type, follow-up, and treatment context [2,10,11].

Follow-up is a central limitation of the present analysis. Observation ranged from 3 to 24 months and was not standardized. Patients with recurrence had longer available follow-up than those without documented recurrence, indicating greater opportunity for recurrence detection. In addition, 72/214 patients had no documented follow-up, and these patients differed from the followed patients in sex, treatment modality, and overall diagnosis distribution. This differential attrition means that the recurrence proportion cannot be assumed to represent the entire original cohort. For this reason, recurrence is reported only among patients with documented initial lesion resolution and the total cohort is not used as a recurrence denominator.

Smoking was not significantly associated with recurrence. Although the point estimate was above unity (OR 1.65), the 95% CI was wide (0.67–4.08) and compatible with little or no association as well as with an increased risk. The present dataset therefore does not provide evidence that smoking predicts recurrence. Tobacco exposure remains clinically relevant to laryngeal mucosal health. Structural alterations in Reinke edema have been described histopathologically [12], while smoking-related voice changes in this condition have also been investigated [13]. These broader observations should not be conflated with a recurrence effect demonstrated by this study.

Documented reflux disease/LPR and professional voice use were also not significantly associated with recurrence. These exposures were retrospectively coded as broad categorical variables, without objective reflux testing or quantitative measures of vocal load. Formal voice therapy was not systematically available locally, and self-reported use of independent voice training or therapy was not consistently documented. Consequently, the influence of voice therapy, adherence, reflux control, changes in vocal behavior, or smoking cessation on persistence or recurrence could not be evaluated.

The comparison between cold-instrument and CO_2_ laser surgery was deliberately exploratory. Recurrence was numerically higher after cold-instrument surgery (24.1%) than after CO_2_ laser surgery (11.1%), but the difference was not statistically significant and the confidence interval was wide. The technique groups also differed markedly in lesion composition, with all followed vascular ectasia and Reinke edema surgical cases occurring in the cold-instrument group. This supports substantial confounding by indication. A polyp-only sensitivity analysis produced a similar direction of effect but remained non-significant and imprecise. These findings are therefore hypothesis-generating and cannot establish comparative effectiveness or superiority of either technique.

Potential technical advantages and disadvantages of the two surgical approaches described in the literature should be distinguished from the findings of this study. Conventional cold instruments provide tactile dissection and avoid thermal energy, whereas CO_2_ laser surgery can provide precise cutting and hemostasis when conservative settings are used [14]. Conversely, thermal spread is a recognized concern with laser surgery. The present study did not measure operative complications, postoperative edema, scarring, validated voice outcomes, mucosal-wave recovery, or other functional endpoints; no conclusions regarding these outcomes can be drawn from our data.

Systematic videostroboscopy was not available. This limitation affects not only baseline diagnostic characterization but also the sensitivity and consistency of recurrence detection, because subtle residual or recurrent lesions may be missed on routine laryngeal examination. Narrow-band imaging may also provide additional vascular information in selected mucosal lesions [15], but it was not part of a standardized follow-up protocol in this cohort.

The laterality analysis should also be interpreted according to the database definition. The recorded variable represented the dominant/index lesion. In patients with a dominant vocal fold nodule and a contralateral reactive/contact lesion, only the dominant side was coded. Accordingly, the finding that the dominant/index side was not significantly associated with recurrence does not represent a comparison of all true unilateral versus bilateral disease.

### 4.1. Clinical Implications

The principal clinical finding is that recurrence was documented in 17.0% of patients after confirmed initial lesion resolution during non-standardized real-world follow-up. This supports the clinical value of continued surveillance, while the present data do not define an optimal follow-up schedule. No individual demographic or clinical factor demonstrated a statistically significant association with recurrence, and this study therefore does not show that smoking cessation, reflux treatment, vocal hygiene, or voice therapy prevent recurrence. These measures remain reasonable components of broader voice-care practice, but their effects on recurrence were not established here. Systematic documentation of lesion resolution, follow-up duration, surgical technique, voice therapy exposure, and longitudinal risk-factor modification should be incorporated into future clinical databases.

### 4.2. Limitations

Several limitations should be acknowledged. First, the retrospective single-center design introduces selection and information bias and limits generalizability. Second, follow-up was not protocolized: 72 patients had no documented follow-up, and among the followed patients, observation ranged from 3 to 24 months. Patients with recurrence had longer observation, and patients without follow-up differed in sex, treatment modality, and overall diagnosis distribution, demonstrating potential ascertainment and attrition bias. Exact time-to-recurrence dates were not consistently available, so time-to-event analysis could not be performed.

Third, although the source-record review allowed persistent lesions to be separated from recurrences according to documented initial resolution, no standardized minimum disease-free interval had been prespecified. Fourth, systematic videostroboscopy was unavailable for baseline and follow-up examinations, which may have affected both lesion classification and recurrence detection. Fifth, smoking, alcohol exposure, reflux status, professional voice use, longitudinal changes in these factors, and adherence to vocal hygiene recommendations were incompletely quantified. Formal voice therapy exposure, timing, number of sessions, and adherence could not be analyzed because these services were not systematically available locally and self-reported external therapy was not consistently recorded.

Finally, surgical technique was not randomized and was influenced by lesion type, surgeon preference, equipment availability, and intraoperative assessment. The followed technique groups differed in lesion composition, creating substantial potential for confounding by indication. Only 10 recurrences occurred among the 56 followed surgical patients, and only eight events occurred in the polyp-only sensitivity analysis. These sample sizes precluded a reliable, adjusted comparison and substantially limited statistical power.

### 4.3. Future Directions

Prospective multicenter studies should use a standardized definition that explicitly distinguishes persistence from recurrence, require documented lesion resolution, and use predefined follow-up intervals with exact time-to-event recording. Systematic videostroboscopy, validated voice outcome instruments, quantitative smoking and vocal-load measures, objective or standardized reflux assessment, documented voice therapy exposure, lesion-size grading, and standardized patient-level surgical technique parameters would improve comparability. Larger technique-specific cohorts are needed to permit adjustment for lesion subtype and other confounders and to determine whether selected lesions benefit from one surgical approach over another.

## 5. Conclusions

In this retrospective cohort of 214 adults with benign vocal fold lesions, follow-up was documented in 142 patients. After separating persistent disease from recurrence, 23 of 135 patients with documented initial lesion resolution developed recurrence (17.0%), while seven patients had persistent disease at the initial post-treatment assessment. No evaluated clinical factor showed a statistically significant association with recurrence in univariate analysis. Among the followed surgical patients, recurrence was numerically lower after CO_2_ laser surgery than after cold-instrument surgery, but the difference was not statistically significant and cannot establish superiority because of non-random treatment selection, lesion heterogeneity, limited events, and potential confounding by indication. The recurrence proportion should also be interpreted in light of variable follow-up and differential attrition.

## Figures and Tables

**Table 1:** Cohort characteristics, follow-up status, and outcome classification.

Variable	Total Cohort (n = 214)
Age, mean ± SD	49.6 ± 16.9
Age, median (range)	52 (18–80)
Male sex	138 (64.5%)
Female sex	76 (35.5%)
Urban residence	173 (80.8%)
Rural residence	41 (19.2%)
Professional voice user	85 (39.7%)
Current smoker	77 (36.0%)
Chronic alcohol use	39 (18.2%)
Reflux disease/LPR	112 (52.3%)
Surgical treatment	103 (48.1%)
Conservative treatment	111 (51.9%)
Documented follow-up	142 (66.4%)
No documented follow-up	72 (33.6%)
Documented initial lesion resolution	135/142 (95.1%)
Persistent disease at initial assessment	7/142 (4.9%)
Documented recurrence after initial resolution	23/135 (17.0%)
No documented recurrence during available follow-up	112/135 (83.0%)

SD, standard deviation; LPR, laryngopharyngeal reflux.

**Table 2:** Distribution of benign vocal fold lesions in the total cohort.

Diagnosis	N	%
Vocal fold polyp	92	43.0
Vocal fold nodule	87	40.7
Reinke edema	14	6.5
Vascular ectasia	10	4.7
Post-intubation granuloma	4	1.9
Sulcus glottidis	4	1.9
Intracordal cyst	3	1.4

**Table 3:** Laterality of the dominant/index lesion in the total cohort.

Dominant/Index Lesion Laterality	n	%
Left	102	47.7
Right	94	43.9
Bilateral	18	8.4

Note: In cases with a dominant lesion and a contralateral reactive/contact lesion, the laterality field reflected the dominant/index lesion only.

**Table 4:** Documented recurrence according to lesion type among patients with documented initial lesion resolution.

Diagnosis	Initial Resolution, n	Documented Recurrences, n	Recurrence Proportion (%)
Vocal fold polyp	53	10	18.9
Vocal fold nodule	61	11	18.0
Reinke edema	8	2	25.0
Vascular ectasia	5	0	0.0
Post-intubation granuloma	3	0	0.0
Sulcus glottidis	3	0	0.0
Intracordal cyst	2	0	0.0
Total	135	23	17.0

**Table 5:** Univariate associations with documented recurrence among patients with documented initial lesion resolution (n = 135).

Characteristic	Recurrence (n = 23)	No Documented Recurrence (n = 112)	OR (95% CI)	*p*-Value
Age, mean ± SD, years	50.4 ± 17.9	49.2 ± 16.3	-	0.761
Male sex	15 (65.2%)	67 (59.8%)	1.26 (0.49–3.22)	0.815
Urban residence	20 (87.0%)	92 (82.1%)	1.45 (0.39–5.35)	0.764
Professional voice user	11 (47.8%)	46 (41.1%)	1.32 (0.53–3.24)	0.645
Current smoker	11 (47.8%)	40 (35.7%)	1.65 (0.67–4.08)	0.346
Chronic alcohol use	5 (21.7%)	21 (18.8%)	1.20 (0.40–3.61)	0.773
Reflux disease/LPR	13 (56.5%)	61 (54.5%)	1.09 (0.44–2.69)	1.000
Surgical treatment	10 (43.5%)	46 (41.1%)	1.10 (0.45–2.73)	0.821

Age was compared using Welch’s *t*-test; categorical variables were compared using Fisher’s exact test. ORs are unadjusted exploratory estimates. SD, standard deviation; OR, odds ratio; CI, confidence interval; LPR, laryngopharyngeal reflux.

**Table 6:** Baseline characteristics of the followed surgical analytic cohort according to technique.

Characteristic	Cold Instruments (n = 29)	CO_2_ Laser (n = 27)
Age, mean ± SD, years	45.8 ± 17.1	47.8 ± 16.7
Male sex	18 (62.1%)	16 (59.3%)
Professional voice user	9 (31.0%)	8 (29.6%)
Current smoker	13 (44.8%)	10 (37.0%)
Chronic alcohol use	9 (31.0%)	8 (29.6%)
Reflux disease/LPR	15 (51.7%)	13 (48.1%)
Vocal fold polyp	19 (65.5%)	26 (96.3%)
Reinke edema	4 (13.8%)	0 (0.0%)
Vascular ectasia	5 (17.2%)	0 (0.0%)
Intracordal cyst	1 (3.4%)	1 (3.7%)

SD, standard deviation; LPR, laryngopharyngeal reflux.

**Table 7:** Exploratory recurrence analyses according to surgical technique.

Analysis	Cold Instruments	CO_2_ Laser	OR (95% CI)	Fisher *p*
All followed surgical patients	7/29 (24.1%)	3/27 (11.1%)	2.55 (0.58–11.08)	0.299
Polyp-only sensitivity analysis	5/19 (26.3%)	3/26 (11.5%)	2.74 (0.57–13.27)	0.253

OR, odds ratio; CI, confidence interval.

**Table 8:** Follow-up duration according to outcome and treatment subgroup.

Comparison	Group 1 Follow-Up	Group 2 Follow-Up	Mann–Whitney U *p*
Documented recurrence (n = 23) vs. no documented recurrence (n = 112)	11.6 ± 5.9 mo; median 12 (3–24)	8.7 ± 4.5 mo; median 6 (3–24)	0.016
Surgical (n = 56) vs. conservative management (n = 79)	10.2 ± 5.4 mo; median 9 (6–24)	8.5 ± 4.3 mo; median 6 (3–24)	0.114
Cold instruments (n = 29) vs. CO_2_ laser (n = 27)	11.3 ± 6.4 mo; median 9 (6–24)	9.0 ± 4.0 mo; median 6 (6–18)	0.247

Values are mean ± SD; median (range). Follow-up was measured to the last documented laryngeal examination and was not protocolized. SD, standard deviation; mo, months.

**Table 9:** Baseline comparison of patients with and without documented follow-up.

Characteristic	Documented Follow-Up (n = 142)	No Documented Follow-Up (n = 72)	*p*-Value
Age, mean ± SD, years	49.5 ± 16.8	49.7 ± 17.2	0.958
Male sex	83 (58.5%)	55 (76.4%)	0.010
Urban residence	118 (83.1%)	55 (76.4%)	0.271
Professional voice user	60 (42.3%)	25 (34.7%)	0.304
Current smoker	54 (38.0%)	23 (31.9%)	0.452
Chronic alcohol use	26 (18.3%)	13 (18.1%)	1.000
Reflux disease/LPR	79 (55.6%)	33 (45.8%)	0.194
Surgical treatment	56 (39.4%)	47 (65.3%)	<0.001
Vocal fold polyp	53 (37.3%)	39 (54.2%)	0.042 *
Vocal fold nodule	66 (46.5%)	21 (29.2%)	
Reinke edema	8 (5.6%)	6 (8.3%)	
Vascular ectasia	5 (3.5%)	5 (6.9%)	
Post-intubation granuloma	4 (2.8%)	0 (0.0%)	
Sulcus glottidis	4 (2.8%)	0 (0.0%)	
Intracordal cyst	2 (1.4%)	1 (1.4%)	
Cold-instrument technique among surgical patients	29/56 (51.8%)	26/47 (55.3%)	0.843

Age was compared using Welch’s *t*-test; binary variables were compared using Fisher’s exact test. * Overall Fisher–Freeman–Halton exact test for diagnosis distribution; individual lesion-specific *p*-values were not calculated because of sparse cells. The surgical technique comparison is restricted to surgically treated patients. SD, standard deviation; LPR, laryngopharyngeal reflux.

## Data Availability

The data supporting the findings of this study are available from the corresponding author upon reasonable request. The dataset is not publicly available because it contains clinical information subject to institutional, ethical, and privacy restrictions.

## References

[1] Stachler R.J., Francis D.O., Schwartz S.R., Damask C.C., Digoy G.P., Krouse H.J., McCoy S.J., Ouellette D.R., Patel R.R., Reavis C.C.W. (2018). Clinical Practice Guideline: Hoarseness (Dysphonia) (Update). Otolaryngol. Head Neck Surg..

[2] Gocal W.A., Tong J.Y., Maxwell P.J., Sataloff R.T. (2025). Systematic Review of Recurrence Rates of Benign Vocal Fold Lesions Following Surgery. J. Voice.

[3] Bohlender J. (2013). Diagnostic and therapeutic pitfalls in benign vocal fold diseases. GMS Curr. Top. Otorhinolaryngol. Head Neck Surg..

[4] de Vasconcelos D., Gomes A.O.C., de Araújo C.M.T. (2019). Vocal Fold Polyps: Literature Review. Int. Arch. Otorhinolaryngol..

[5] Lechien J.R., Saussez S., Nacci A., Barillari M.R., Rodriguez A., Le Bon S.D., Crevier-Buchman L., Harmegnies B., Finck C., Akst L.M. (2019). Association between laryngopharyngeal reflux and benign vocal folds lesions: A systematic review. Laryngoscope.

[6] Malik P., Yadav S.P.S., Sen R., Gupta P., Singh J., Singla A., Vashisht S. (2019). The Clinicopathological Study of Benign Lesions of Vocal Cords. Indian J. Otolaryngol. Head Neck Surg..

[7] Rosen C.A., Gartner-Schmidt J., Hathaway B., Simpson C.B., Postma G.N., Courey M., Sataloff R.T. (2012). A nomenclature paradigm for benign midmembranous vocal fold lesions. Laryngoscope.

[8] Mansuri B., Tohidast S.A., Soltaninejad N., Kamali M., Ghelichi L., Azimi H. (2018). Nonmedical Treatments of Vocal Fold Nodules: A Systematic Review. J. Voice.

[9] Ogawa M., Inohara H. (2018). Is voice therapy effective for the treatment of dysphonic patients with benign vocal fold lesions?. Auris Nasus Larynx.

[10] Lee M., Sulica L. (2020). Recurrence of benign phonotraumatic vocal fold lesions after microlaryngoscopy. Laryngoscope.

[11] Patrial M.T.C.R.O., Hamerschmidt R., Matias J.E.F., Macedo Filho E.D., Carvalho B. (2021). Analysis of Surgical Recurrence after Larynx Microsurgery for Benign Lesions. Int. Arch. Otorhinolaryngol..

[12] Sakae F.A., Imamura R., Sennes L.U., Tsuji D.H., Mauad T., Saldiva P.H.N. (2010). Elastic fibers in Reinke’s edema. Ann. Otol. Rhinol. Laryngol..

[13] Martins R.H.G., Tavares E.L.M., Pessin A.B.B. (2017). Are Vocal Alterations Caused by Smoking in Reinke’s Edema in Women Entirely Reversible After Microsurgery and Smoking Cessation?. J. Voice.

[14] Kumar S., Prasad B.K. (2019). A Comparison of Surgical Outcomes of Carbon Dioxide Laser Versus Conventional Cold Instrument Excision of Benign Vocal Cord Lesions. Indian J. Otolaryngol. Head Neck Surg..

[15] Davaris N., Voigt-Zimmermann S., Roessner A., Arens C. (2017). Narrow band imaging for evaluation of laryngeal mucosal lesions. HNO.

